# Rhizosphere frame system enables nondestructive live-imaging of legume-rhizobium interactions in the soil

**DOI:** 10.1007/s10265-023-01476-2

**Published:** 2023-07-04

**Authors:** Hanna Nishida, Yoshikazu Shimoda, Khin Thuzar Win, Haruko Imaizumi-Anraku

**Affiliations:** Institute of Agrobiological Sciences, National Agriculture and Food Research Organization, 3-1-3 Kannondai, Tsukuba, Ibaraki 305-8604 Japan

**Keywords:** Fluorescence stereomicroscope, Live imaging, Nondestructive observation, Plant–microbe interaction, Rhizobium, Root nodule symbiosis

## Abstract

Most plants interact with various soil microorganisms as they grow through the soil. Root nodule symbiosis by legumes and rhizobia is a well-known phenomenon of plant–microbe interactions in the soil. Although microscopic observations are useful for understanding the infection processes of rhizobia, nondestructive observation methods have not been established for monitoring interactions between rhizobia and soil-grown roots. In this study, we constructed *Bradyrhizobium diazoefficiens* strains that constitutively express different fluorescent proteins, which allows identification of tagged rhizobia by the type of fluorophores. In addition, we constructed a plant cultivation device, Rhizosphere Frame (RhizoFrame), which is a soil-filled container made of transparent acrylic plates that allows observation of roots growing along the acrylic plates. Combining fluorescent rhizobia with RhizoFrame, we established a live imaging system, RhizoFrame system, that enabled us to track the nodulation processes with fluorescence stereomicroscope while retaining spatial information about roots, rhizobia, and soil. Mixed inoculation with different fluorescent rhizobia using RhizoFrame enabled the visualization of mixed infection of a single nodule with two strains. In addition, observation of transgenic *Lotus japonicus* expressing auxin-responsive reporter genes indicated that RhizoFrame system could be used for a real-time and nondestructive reporter assay. Thus, the use of RhizoFrame system is expected to enhance the study of the spatiotemporal dynamics of plant–microbe interactions in the soil.

**Supplementary Information:**

The online version contains supplementary material available at 10.1007/s10265-023-01476-2.

## Introduction

The life of most plants begins when their seeds come into contact with the soil. The roots, the organs that interact with the soil and absorb water and nutrients, are affected by the soil’s physical, chemical, and biological environment as they grow through it (Bengough et al. 2011; Karlova et al. 2021; Morris et al. 2017). In particular, the biological environment encompasses interactions with various soil microorganisms, including symbiotic bacteria, such as rhizobia (Lambers et al. 2009). The plant–microbe interactions we observed in the laboratory were conducted under conditions in which the above interactions with the soil environment were eliminated (i.e., the soil was washed off the roots and “cleaned” for microscopic observation). Therefore, we have not been able to fully observe the spatiotemporal information lost in this cleaning process, such as the behavior of microorganisms in the vicinity of root hair cells in contact with the soil, microbial colonization on the root surface, and shifting from colonization to invasion into host plants.

Root nodule symbiosis between legumes and rhizobia is a representative phenomenon of plant–microbe interactions. To establish root nodule symbiosis, rhizobia in the soil must accommodate themselves within nodule cells. Sensing a signal from rhizobia, host plants deform root hairs to trap rhizobia and form tube-like structures called infection threads for bacterial invasion into inner root tissues. Rhizobia divide and grow inside the infection threads while, simultaneously, the dedifferentiation of root cortical cells is induced, and they start dividing to form the nodule. Infection threads extend through the host root hairs toward the developing nodule primordia. Eventually, rhizobia are released from the infection threads and enter the cytoplasm of the inner cells in the nodules. Finally, colonizing rhizobia in the nodule cells fix atmospheric nitrogen into ammonia (Kouchi et al. 2010; Oldroyd 2013; Roy et al. 2020).

Microscopic observations of tagged rhizobia by the expression of fluorescent proteins have facilitated our understanding of these infection processes (Fournier et al. 2008; Gage 2002; Gage et al. 1996; Ledermann et al. 2015; Stuurman et al. 2000). However, previous research has been done primarily using the destructive method of digging roots out of the soil. Cultivation on agar plates is sometimes used for time-lapse imaging of rhizobial infection. However, such experimental conditions cannot fully reproduce the legume–rhizobium interaction dynamics occurring in the soil.

Plants change their root growth pattern to efficiently absorb water and nutrients from the soil (Lynch 1995). In the studies of root phenotype plasticity in response to environmental changes, nondestructive measurement techniques have been developed to observe changes in root systems over time without disrupting the spatial patterns of the roots in the soil. For example, 3D imaging methods with X-ray computed tomography and magnetic resonance imaging allow visualization of roots in soil (Mooney et al. 2012; Rascher et al. 2011; Teramoto et al. 2020; van Dusschoten et al. 2016). On the other hand, to observe roots growing adjacent to the culture vessel surface, plant cultivation in soil-filled transparent boxes or tubes is widely used (Bengough et al. 2016; Huck and Taylor 1982; Jeudy et al. 2016; Neufeld et al. 1989; Zhao et al. 2022). These nondestructive methods primarily focus on measuring root size and architecture. As a recent example, the GLO-Root system has visualized the structure and gene expression patterns of soil-grown roots using plants that express luminescence-based reporters (Rellán-Álvarez et al. 2015). They also observed *Pseudomonas* colonization on Arabidopsis roots at the root system scale level. On the other hand, observations at the microscopic-scale are necessary to understand the dynamics of microbial infection to the host plant roots. As another example, microscopic live imaging of arbuscular mycorrhizal fungi infection of soil-grown roots was performed by using fluorescent reporter rice plants (Kobae and Fujiwara 2014). This method indirectly showed the infection dynamics of arbuscular mycorrhiza fungi by visualizing plant molecular markers.

The current study aimed to develop a nondestructive observation system for the live imaging of legume–rhizobium interactions by fluorescence stereomicroscope in the soil. First, we constructed new strains of *Bradyrhizobium diazoefficiens* USDA 110, which constitutively expressed fluorescent proteins and are suitable for competitive infection analysis of rhizobia. Second, we constructed a plant cultivation device, Rhizosphere Frame (RhizoFrame), by optimizing the size and thickness of the rhizotron for stereomicroscope observation. By combining these tools, we developed RhizoFrame system, which enabled us to visualize the contact of roots and rhizobia in soil and track the nodulation processes at the stereomicroscope level, while retaining spatial information about roots, rhizobia, and soil.

## Materials and methods

### Construction of plasmids and fluorescence-tagged *B. diazoefficiens* strains

The primers used for PCR are listed in Table S1. For the constitutive expression of fluorescent proteins, the promoter region of the *groEL4* gene (blr5626) of *B*. *diazoefficiens* USDA110 (GenBank accession BA000040.2; genome position 6,186,045–6,186,254) was amplified by PCR from *pBjGroEL4::DsRed* construct (Hayashi et al. 2014). *ZsGreen* was optimized and synthesized according to the codon frequency of *Bradyrhizobium* (Fig. S1). *DsRed* was amplified by PCR from the *pBjGroEL4::DsRed* construct (Hayashi et al. 2014), and *tdTomato* was purchased from Takara Bio. Each fluorescent gene was amplified by PCR with primers containing the *trpA* terminator (Wu and Platt 1978) in the reverse primer. *GroEL4* promoter and each fluorescent gene were inserted into the EcoRI site of the pK18mobsacB vector (Schäfer et al. 1994), in which the spectinomycin/streptomycin resistance gene replaced the kanamycin resistance gene.

For chromosomal integration, the downstream region of *nifX* of *B*. *diazoefficiens* USDA110 (GenBank accession BA000040.2; genome position 1,914,497–1,915,496) was amplified by PCR and inserted into the XbaI site of the pK18mobsacB vector. Resultant plasmids were transferred to WT *B. diazoefficiens* USDA110 by triparental mating using pRK2013 as a helper plasmid (Figurski and Helinski 1979). Single crossover transconjugants were selected by their fluorescence and antibiotic resistance to polymyxin and spectinomycin/streptomycin. Integration of plasmid downstream of the *nifX* gene was confirmed by PCR and sequencing.

### Plant growth conditions for the analysis of nodulation phenotype

*B. diazoefficiens* were grown at 28 °C with reciprocal shaking at 140 rpm in HM salt medium (Cole and Elkan 1973) supplemented with 0.1% (w/v) L-arabinose and 0.025% (w/v) Bacto Yeast Extract. Soybeans (*Glycine max* (L.) Merr. cv. Enrei) were grown under a 16 h light/8 h dark cycle at 25 °C in 300 ml pots (CUL-JAR300, IWAKI) containing sterile vermiculite with Broughton and Dilworth (B&D) solution (Broughton and Dilworth 1971) and *B. diazoefficiens* USDA 110 WT or fluorescent strains. After 21 days, the nodule number, nodule dry weight, and acetylene reduction activity (ARA) were determined per plant.

### Acetylene reduction assay

The nitrogen fixation rate was estimated using an acetylene reduction assay (Hardy et al. 1968). Briefly, root systems, including the nodules, were cut off from shoots and incubated in 100 ml glass vials sealed with rubber stoppers, from which 10% (v/v) air was replaced with pure acetylene. The samples were incubated at 25 °C for 20 min to convert acetylene into ethylene. One ml of headspace was injected into a gas chromatograph (GC-2014 SHIMADZU, Kyoto, Japan) equipped with a flame ionization detector. Ethylene was quantified by comparison to the standard curve of pure ethylene, and the values were normalized for injection volumes and incubation times.

### Identification of rhizobia in nodules

Nodules were sterilized using 5% (v/v) sodium hypochlorite solution for 3 min and washed 10 times with sterile distilled water. Total DNA was extracted from nodules as described previously (Shiro et al. 2016). Each nodule was homogenized in 100 μl of sterile distilled water and then 24.5 μl homogenate was treated with 50 μl of BL buffer (Shiro et al. 2016) supplemented with 0.5 μl proteinase K (1 mg ml^–1^). The mixture was incubated at 60 °C for 20 min and 95 °C for 5 min. After centrifugation, the supernatant was used as the PCR template. PCR was performed using a GoTaq G2 Hot Start Green Master Mix (Promega) according to the manufacturer’s protocol. The primers used for PCR are listed in Table S1.

### Commercially available materials for making handmade RhizoFrame

The following materials were used to assemble handmade RhizoFrame (Fig. S2).

Transparent acrylic plates: 100 mm wide, 1 mm thick, and 150 mm high, custom-made. The bottom part of the acrylic plates need not be rounded. When rounding off the corners, process with R = 15.0 mm.

Spacer: 6 mm wide and 3 mm thick, NICHIAS SOFT SEAL (NICHIAS Co.)

Water-resistant double-sided tape: 5 mm wide and 0.8 mm thick, High Tack Double-Sided Adhesive Tape (3 M Japan Limited).

Polyester mesh: 38 μm aperture, cut into 5 × 4 cm.

Waterproof tape: Ace Cloth 011 Single-Sided Airtight Waterproof Tape (Koyo Chemical).

### Plant growth using RhizoFrame

*Mesorhizobium loti* was grown at 28 °C with reciprocal shaking at 140 rpm in a YEM medium. RhizoFrame was filled with about 75 ml of the soil. Twenty-five ml B&D solution containing 1 × 10^5^ rhizobia was poured over the soil. RhizoFrame was covered with a stainless steel cover and allowed to stand for 3 days at 25 °C. Soybeans (*G. max* cv. Fukuyutaka or Enrei) were germinated in sterile vermiculite at 25 °C for 2 days in darkness and then transferred in RhizoFrame containing sterile vermiculite or heat-processed granular soil (fertilizer-free soil, Kanuma Sangyo Co.) with *B. diazoefficiens* USDA 110. RhizoFrame with a stainless steel covering was placed in a water reservoir to moisten the soil. Plants in RhizoFrame were grown under a 16 h light/8 h dark cycle at 25 °C. *Lotus japonicus* (Regel) K.Larsen (Miyakojima MG-20) were germinated in agar plates at 24 °C for the first 2 days in darkness and the next day in a 16 h light/8 h dark cycle and then transferred in RhizoFrame containing sterile vermiculite with *M. loti* MAFF303099.

### Microscopic observations

Microscopy was performed using the on-axis zoom microscope Axio Zoom V16 (ZEISS). GFP and ZsGreen were detected through the GFP filter (ZEISS), and DsRed and tdTomato through the mRFP filter (ZEISS). Images were acquired using the ZEN 3.3 software (ZEISS).

## Results

### Construction of novel *B. diazoefficiens* USDA 110 strains constitutively expressing fluorescent proteins

**Fig. 1:**
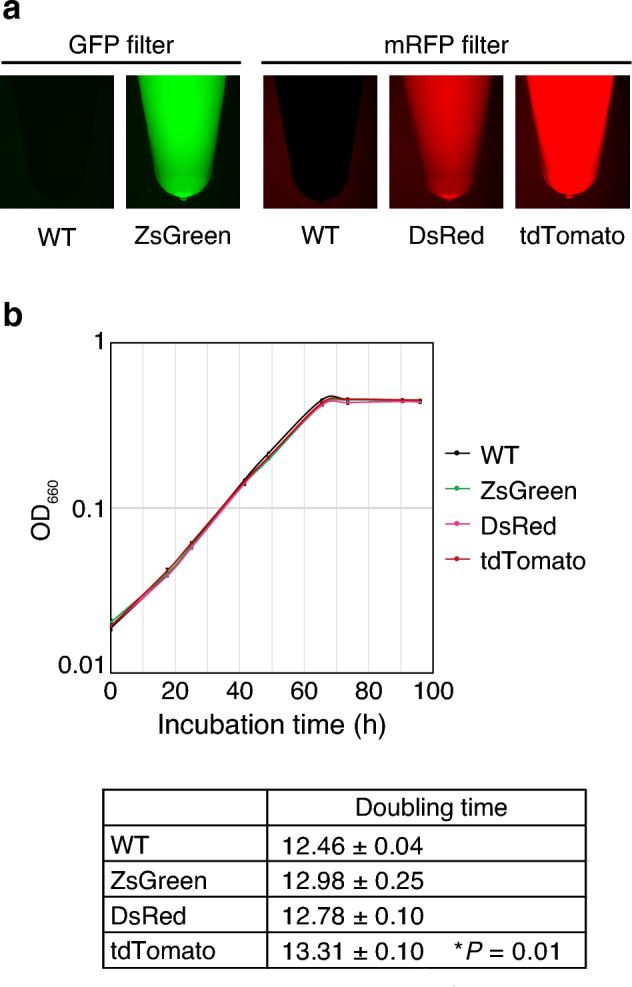
Characteristics of *B. diazoefficiens* USDA 110 strains constitutively expressing each fluorescent protein. **a** Fluorescent images of cell suspension (OD_660_ = 0.5) of WT and each fluorescent rhizobium (ZsGreen, DsRed, and tdTomato). Fluorescence images were acquired under the same exposure time through GFP and mRFP filters, respectively. **b** Growth curve and doubling time ± SE (*n* = 3–4) of WT and each fluorescent rhizobia. Growth was measured by recording the culture medium’s optical density (660 nm). OD_660_ value is plotted versus incubation time (hours: h). The significance of the differences with WT (*P < 0.05) was determined with Dunnett’s test

**Fig. 2:**
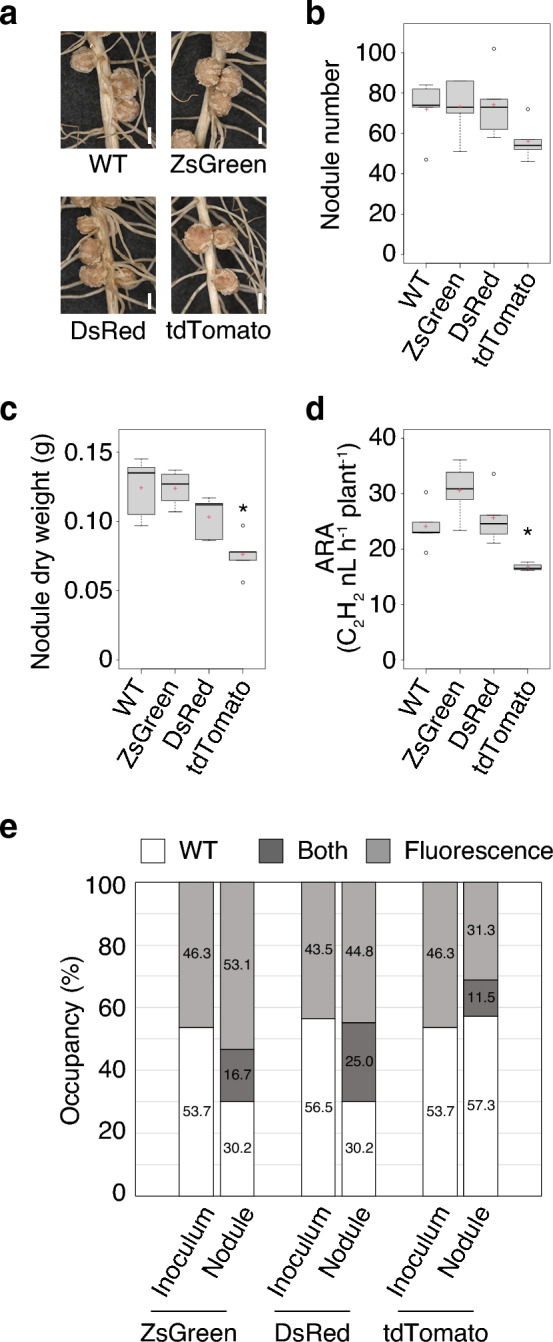
Symbiosis phenotypes at 21 days after inoculation (dai) in soybean cv. Enrei inoculated with *B. diazoefficiens* USDA 110 strains, WT or mutated ones, each constitutively expressing fluorescent proteins ZsGreen, DsRed, or tdTomato. **a** Nodule formation, **b** nodule number (*n* = 5 plants), **c** nodule dry weight (*n* = 5 plants), and **d** acetylene reduction activity (ARA; *n* = 4–5 plants). Scale bars = 2 mm (**a**). In panels **b**–**d**, centerlines in the boxplots show the medians, and upper and lower quartile limits are shown as horizontal bars. Points represent outliers, and red crosses indicate the sample means. The significance of differences with WT was determined with Dunnett’s test (*P < 0.05). **e** Nodule occupancy during co-inoculation of WT with each fluorescent rhizobia (ZsGreen, DsRed, and tdTomato; *n* = 4 plants, 96 nodules). Enrei was inoculated with approximately 1:1 mixture of WT and fluorescent rhizobia, and after 21 days, the number of nodules colonized by each strain was measured. Each strain in nodules was identified by PCR. “Inoculum” indicates the ratio of colonies formed by rhizobia on agar after mixture was plated (*n* = 745–858 colonies)

To visualize soybean-rhizobium interactions, we constructed soybean symbiont *B. diazoefficiens* USDA 110 strains that constitutively express each of the three fluorescent proteins: ZsGreen, DsRed, and tdTomato (Fig. 1a). To ensure high fluorescence intensities, each fluorescent protein was positioned downstream of the *BjgroEL4* promoter, which is constitutively expressed in rhizobia (Babst et al. 1996; Fischer et al. 1993; Hayashi et al. 2014). It has been reported that plasmids tend to be rapidly lost in the absence of antibiotic selection in *B. diazoefficiens* (Stuurman et al. 2000). Therefore, we integrated the fluorescent protein-encoding genes into the chromosome of *B. diazoefficiens* USDA110 by homologous recombination. We integrated a fluorescent protein gene into the downstream of *nifX*, a part of the conserved cluster of *nif* genes responsible for nitrogen fixation (Hennecke 1990) and is not expected to be related to the infection competence of the rhizobia. Fluorescent rhizobia exhibited different fluorescence intensities depending on the type of fluorophores. In the red fluorescent proteins, tdTomato strain showed the stronger fluorescence intensity than DsRed (Fig. 1a). To determine the effect of the integration of fluorescent proteins on rhizobia growth, we measured the doubling time of each fluorescent rhizobia (Fig. 1b). The doubling times of the ZsGreen and DsRed strains were the same as that of the wild-type (WT) USDA110. In contrast, the tdTomato strain had a slightly longer doubling time than WT. Next, to evaluate the effect of the integration of fluorescent proteins on root nodule symbiosis, nodulation phenotypes between soybean cv. “Enrei” and each fluorescent rhizobia were examined (Fig. 2a–d). Plants inoculated with ZsGreen and DsRed strains formed mature nodules (Fig. 2a). Their nodule number, nodule dry weight, and ARA were comparable to those of WT rhizobia-inoculated plants (Fig. 2b–d). The tdTomato strain formed mature nodules on soybeans (Fig. 2a), whereas the symbiotic phenotypes tended to be slightly inferior to WT (Fig. 2b–d). To compare the competitiveness of fluorescent rhizobia against WT, we performed mixed infection tests. Approximately 1:1 mixture of fluorescent rhizobia and WT was inoculated into soybeans. Simultaneously, the mixture of rhizobia was plated onto an agar medium to determine the ratio of rhizobia by colony counting method (data shown as “inoculum” in Fig. 2e). After 21 days, the number of nodules colonized by each strain was counted (Fig. 2e). The percentage of each type of fluorescent rhizobia in nodules was not markedly lower than that of WT. Similarly, the competitiveness between fluorescent rhizobia was examined. The percentage of ZsGreen in nodules was similar to that of DsRed, while nodule occupancy of tdTomato was lower than that of ZsGreen (Fig. S3). These results suggest that both bacterial growth and symbiotic properties were unaffected by the integration of the fluorescent genes downstream of *nifX*. However, the constitutive expression of tdTomato by the *BjgroEL4* promoter seems to have a slight negative effect on rhizobial performance.

### Construction of RhizoFrame for nondestructive observation device of legume–rhizobium interactions

**Fig. 3:**
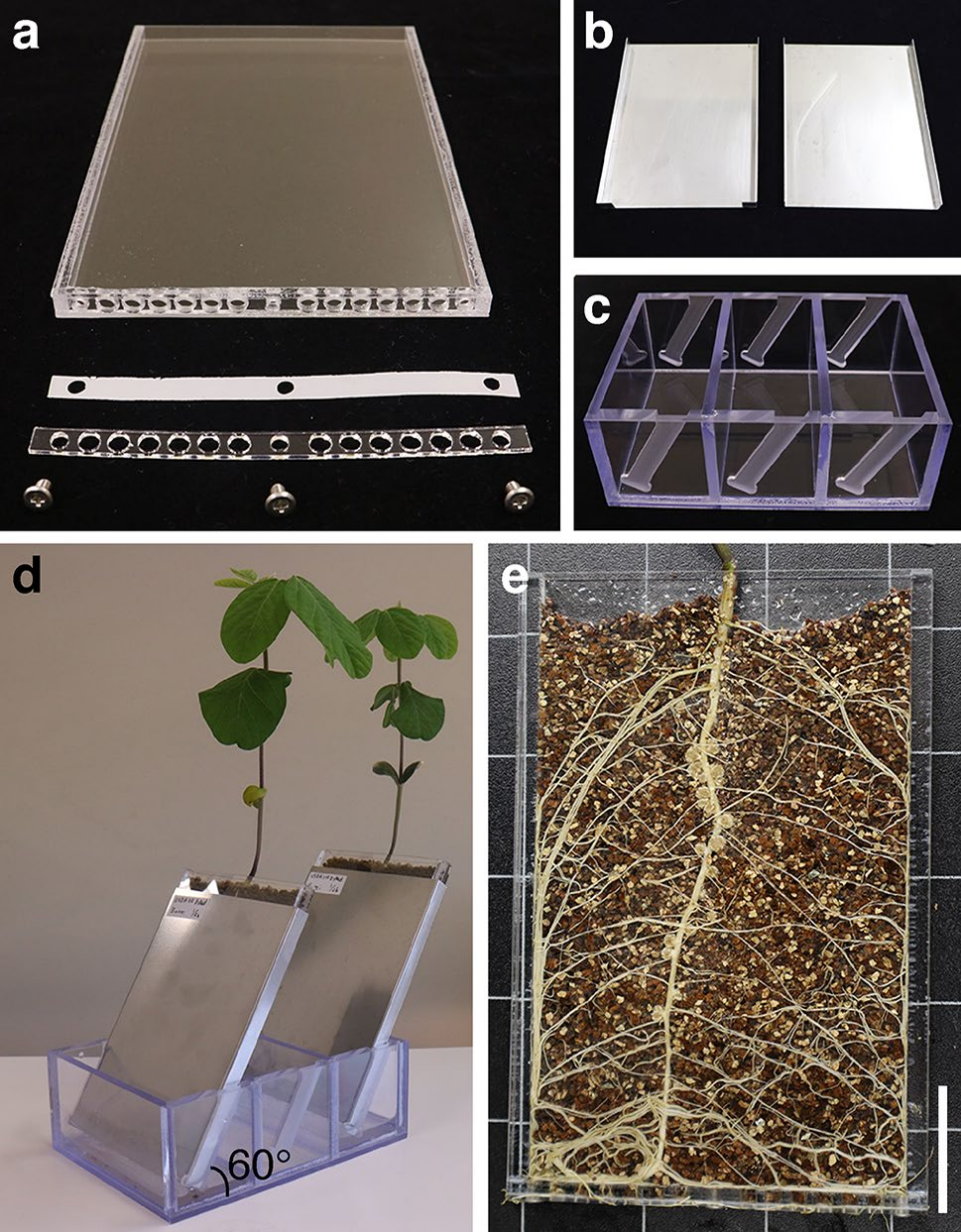
RhizoFrame, a plant cultivation device for visualizing legume–rhizobium interactions. **a** Components of RhizoFrame (from top to bottom: acrylic box, polyester mesh, acrylic plate, and screws). **b** Stainless steel covers for shading RhizoFrame. **c** Water reservoir for standing RhizoFrame. **d** Fourteen-day-old soybeans grown in RhizoFrame, inclined at 60°. **e** Roots and nodules at 14 days after inoculation in RhizoFrame. Scale bars = 3 cm. The assembly of RhizoFrame with commercially available materials is shown in Fig. S2

We modified the size and thickness of the rhizotron to construct RhizoFrame optimized for stereomicroscopic observation (Fig. 3a). RhizoFrame is a container made of 1 mm thick transparent acrylic plates with holes at the bottom. The container is 100 mm wide, 7 mm thick, and 150 mm high, with 5 mm of space between the acrylic plates to add the soil. Stainless steel covers were attached to the outside of RhizoFrame to protect roots from light during plant growth (Fig. 3b). RhizoFrame was placed in a water reservoir to tilt it at 60°, which allowed the roots to grow along the acrylic plate (Fig. 3c, d). Water was added to the reservoir to maintain the soil moisture. A polyester mesh (38 μm aperture) was placed between an acrylic plate and the bottom of RhizoFrame to cover the holes, allowing plants to absorb water while keeping roots inside the container (Fig. 3a). In field soil, infection events are initiated when elongating plant roots come into contact with soil. To simulate the field soil environment, we poured a culture solution of rhizobia over the soil in RhizoFrame and allowed 3 days to settle on the soil. Then, two-day-old soybean seedlings were transferred to the rhizobia-settled RhizoFrame (Fig. S4a). At any given time during cultivation, the cover attached to RhizoFrame was removed, and roots were observed using a microscope (Fig. S4b). RhizoFrame allowed nondestructive observation of root growth and nodule formation in the soil through an acrylic plate (Fig. 3e). Soybeans grown in RhizoFrame formed many mature nodules (Figs. 3e, S5a), while the number of the nodules was lower than soybeans grown in pots (Fig. S5b).

### Live imaging of root nodule symbiosis in soybeans using RhizoFrame system

**Fig. 4:**
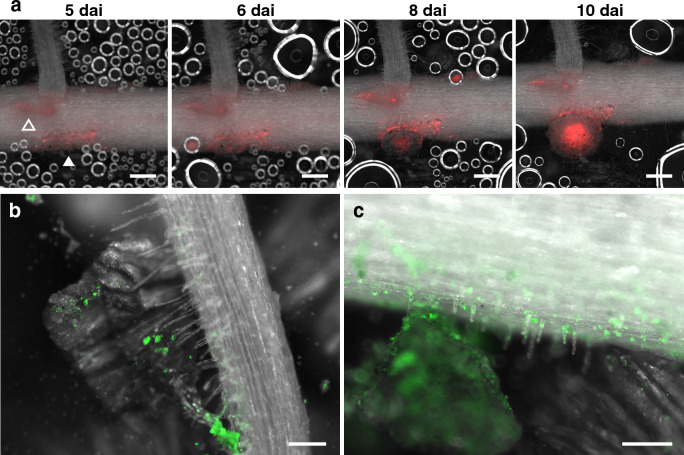
Visualization of soybean-rhizobium interactions using RhizoFrame system. **a** Time-lapse image series of nodulation from 5 to 10 days after inoculation (dai). Arrowheads indicate the root areas where cortical cell division occurs. **b** and **c** A root and vermiculite inoculated with ZsGreen-labeled rhizobium at 7 dai. Fluorescent rhizobia localized on vermiculite particles, the root surface, and root hair cells. Soybean cv. Fukuyutaka (**a**) or Enrei (**b** and **c**) were inoculated with *B. diazoefficiens* USDA 110 strains DsRed (**a**) or ZsGreen (**b** and **c**) and were observed by a fluorescence stereomicroscope. Red and green fluorescences indicate the presence of rhizobia. Scale bars = 500 μm (**a**), 200 μm (**b** and **c**)

We established RhizoFrame system by combining cultivation device (RhizoFrame) with fluorescent rhizobia. Using this system, time-lapse imaging of the dynamics of soybean root nodule symbiosis in the soil was captured by fluorescence stereomicroscopy (Fig. 4a, Movie S1). Five days after inoculation (dai), cortical cell division was observed in the rhizobial colonized zone (Fig. 4a; white arrowhead). The dividing cortical cells led to the initial bulge of the nodule primordia, where the rhizobial invasion was observed (Fig. 4a; 6 dai). The nodule was formed by the enlargement of the nodule primordia in which the rhizobia expanded their infection zone (Fig. 4a; 8 dai, 10 dai). In the area indicated by an open arrowhead (Fig. 4a; 5 dai), rhizobial colonization on epidermal cells and initial cell division were observed, while the formation of nodule primordia ceased (Fig. 4a; 6 dai, 8 dai, 10 dai). We also observed the interactions between soil, roots, and rhizobia (Fig. 4b, c). In RhizoFrame inoculated with fluorescent rhizobia, the rhizobia were observed distributed on the surface of the vermiculite particles, especially in the grooves of the layer structure (Fig. S6a; arrowheads). In RhizoFrame seeded with plants, contact between root hairs and rhizobia-settled vermiculite was observed (Fig. 4b). In addition, colonies of the rhizobia were observed on the epidermal cells of the roots, including root hair cells (Fig. 4c). No green fluorescence was observed on the uninoculated soil (Fig. S6b).

**Fig. 5:**
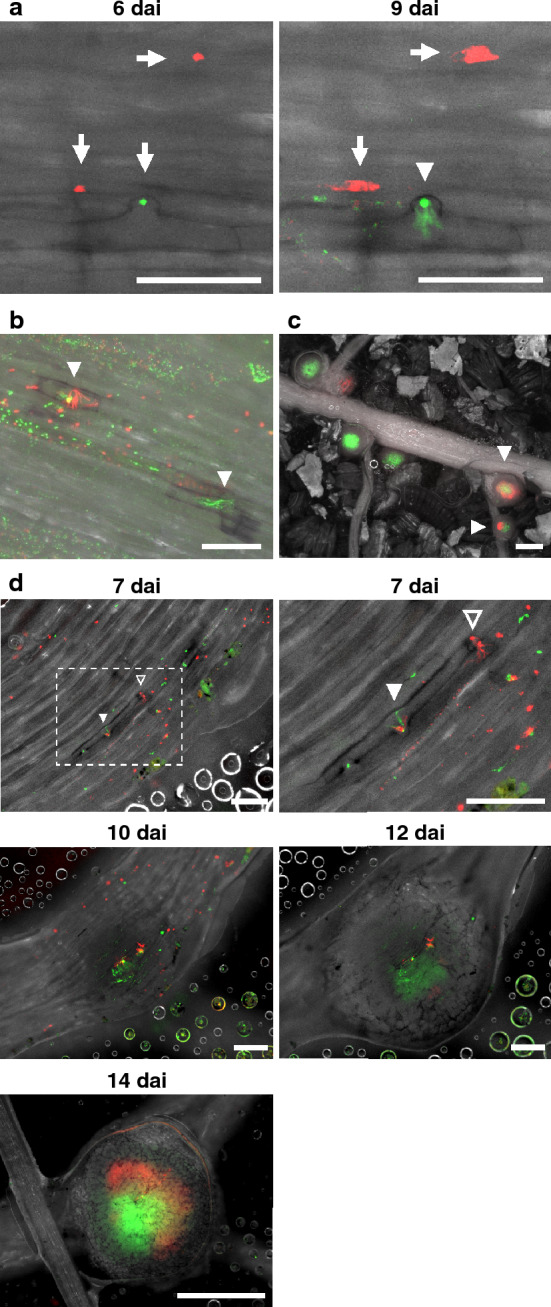
Visualization of nodulation in soybeans inoculated with a mixture of *B. diazoefficiens* USDA110 strains ZsGreen and tdTomato using RhizoFrame system. **a** Microcolonies (6 days after inoculation; dai: arrows), colonies spread on epidermal cells (9 dai: arrows), and invasion of rhizobia into the root hair cell via infection threads (9 dai: arrowhead). **b** Infection threads (white arrows) with pigmentated epidermal cells at 9 dai. Multiple colonies of both strains are dispersed on the root. Pigmentation is observed in epidermal cells where infection threads entered. **c** Root and nodules at 14 dai. The strain of rhizobia colonizing inside nodules can be distinguished by fluorescence. Arrowheads indicate nodules colonized by both strains. **d** Time-lapse image series of nodulation from 7 to 14 dai. The upper right panel is an enlarged view of the dashed line. Arrowheads indicate infection threads. Soybean cv. Enrei was co-inoculated with strains ZsGreen and tdTomato (about 1:1 ratio) and observed by a fluorescence microscope. Green and Red fluorescence indicate the presence of strains ZsGreen and tdTomato, respectively. Scale bars = 100 μm (**a**, **b**, and **d**, 7 dai), 200 μm (**d**, 10 dai and 12 dai), and 1 cm (**c** and **d**, 14 dai)

To assess the infection dynamics among the different fluorescent rhizobial strains, we inoculated soybeans with a 1:1 mixture of USDA110 strains expressing ZsGreen or tdTomato using RhizoFrame. Microcolonies formed on the epidermis and at the tips of short root hairs 6 dai (Fig. 5a; arrows). Soon, infection threads emerged from microcolonies in short root hairs, and the infection threads branched and spread within the root hair cells (Figs. 5a; 9 dai, 5b; arrowheads, S7a–c). In our observation, all infection threads observed on the root epidermis (*n* = 80) were occupied by single fluorescent rhizobial strain (7 dai). We also found that epidermal cells containing infection threads were pigmentated (Figs. 5b, S7). In fact, 85% of epidermal cells, including infected threads (*n* = 80), were observed to be browning in roots 7 dai. Many microcolonies that failed to form infection threads were also observed (Fig. 5a; arrows, 5b). Most of the nodules were colonized by only one of the two fluorescent rhizobial strains, whereas some were infected by both strains (Fig. 5c; arrowheads). To observe the process of simultaneous infection of a single nodule with two strains, we performed time-lapse imaging of the nodulation process (Fig. 5d). An infection thread extended from the microcolony of the ZsGreen strain (white arrowhead) and another infection thread from the tdTomato strain (open arrowhead) was observed in different adjacent root hair cells (Fig. 5d; 7 dai). Invasion of the ZsGreen strain into the nodule primordium was observed from 10 dai; the ZsGreen strain expanded its zone of infection into the center of the nodule until 14 dai. On the other hand, the tdTomato strain acquired an infection zone surrounding the infection zone of the ZsGreen strain at 12 dai, and the infection zone of the tdTomato strain expanded into surrounding the infection zone of the ZsGreen strain at 14 dai (Fig. 5d).

### Live imaging of root nodule symbiosis and auxin distribution patterns in *L. japonicus*

**Fig. 6:**
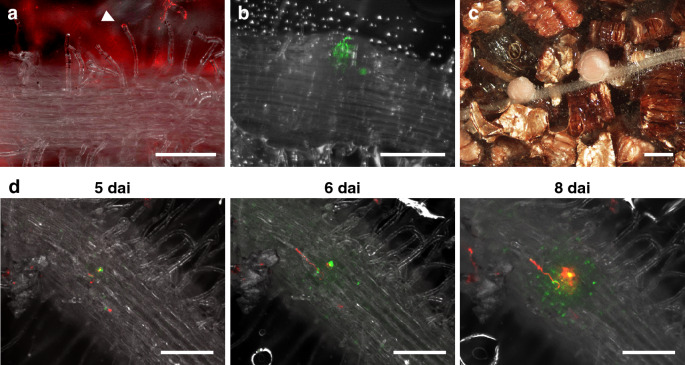
Visualization of nodulation in *L. japonicus* using RhizoFrame system. **a**–**c** Nodulation in *L. japonicus* MG20 plants at 3 days after inoculation (dai: **a**), 7 dai (**b**), and 14 dai (**c**) in RhizoFrame. **a** Curled root hair and infection thread (arrowhead). **b** Branched infection threads invading the nodule primordia. **c** Root and mature nodules. **d** Time-lapse image series of auxin response patterns during nodulation from 5 to 8 dai. Auxin accumulation patterns were shown by GFP signals (green) in *DR5::GFP-NLS* MG20 transgenic plants. Red (**a**, **c**, and **d**) and green (**b**) fluorescence indicate the presence of *M. loti* MAFF303099 that constitutively expresses DsRed or GFP, respectively. Scale bars = 200 μm (**a**, **b**, and **d**) and 1 mm (**c**)

Soybean is a representative crop of the legume family, but its technological development as a target for molecular genetic analysis is still lagging behind. Studies using two model legumes, *L. japonicus* and *Medicago truncatula,* have advanced our understanding of the molecular genetic mechanisms of root symbioses, including root nodule symbiosis (Harrison 2005; Kouchi et al. 2010; Xue et al. 2019). Therefore, we tried time-lapse imaging of nodulation in *L. japonicus* using RhizoFrame system. Similar to the inoculation of *Bradyrhizobium* into soybean, *M. loti* MAFF 303099 expressing DsRed (Maekawa et al. 2009) or GFP (Shimoda et al. 2020) were inoculated on the soil for 3 days before transferring 3-day-old seedlings into RhizoFrame. Because of the smaller plant size of *L. japonicus* than soybean, 4 plants were grown in each RhizoFrame. By 3 dai, curled root hairs and long infection threads growing through them were observed (Fig. 6a), and the branched infected threads invaded the nodule primordia (Fig. 6b). Subsequently, several mature nodules formed on the roots of *L. japonicus* (Fig. 6c). These observations indicate that RhizoFrame system can be used for the nondestructive analysis of the nodulation process in *L. japonicus*.

Next, we examined whether RhizoFrame system could also be used for a reporter assay. As a test case for the reporter assay, we focused on the auxin response during the rhizobial infection. Auxin, one of the most well-known phytohormones, regulates cell proliferation and differentiation during many developmental regulatory processes (Zhao 2010). In root nodule symbiosis, it has been shown that localized auxin accumulation in cortical cells is important for nodule primordium formation (Suzaki et al. 2012). In a previous study, transgenic plants of *L. japonicus* expressing GFP with nuclear localization signal (GFP-NLS) under the control of the auxin-responsive element, *DR5,* were created (Suzaki et al. 2012). In the *DR5::GFP-NLS* transgenic plants, auxin accumulation can be monitored indirectly as GFP expression. To visualize the auxin response during nodulation, we inoculated the *DR5::GFP-NLS* transgenic plants with red fluorescent *M. loti* using RhizoFrame and performed live imaging of auxin accumulation in response to rhizobial infection (Fig. 6d, Movie S2). In roots at 5 dai, GFP was expressed in some cells, and short infection threads were observed in the vicinity of the cells visualized with GFP (Fig. 6d; 5 dai). Subsequently, the infection threads elongated, and cell division progressed with a stronger GFP expression (Fig. 6d; 6 dai). GFP expression was observed in the dividing cortical cells that later become nodule primordia, and rhizobia invaded the dividing cortical cells with a strong GFP signal (Fig. 6d; 8 dai). These observations visualize auxin dynamics synchronized with the rhizobial infection in real time, suggesting that RhizoFrame system can be used for a nondestructive reporter assay.

## Discussion

Tagged rhizobial strains that express fluorescent proteins are a valuable tool for observing bacterial dynamics. If a fluorescent protein is used as a marker for monitoring bacterial activity in plants, the fluorescent protein gene must be stable in the labeled bacterial cells throughout the process of proliferation, colony formation, and infection of the host plants. Plasmid-based overexpression has been applied for the fluorescent tagging of rhizobia (Cheng and Walker 1998; Shimoda et al. 2020; Stuurman et al. 2000). However, in the absence of antibiotic selection, plasmids have been reported to be lost in certain rhizobium species, such as *Bradyrhizobium* (Stuurman et al. 2000). Chromosomal integration of the target genes enables overcoming the problem of plasmid expression and has been successfully employed in *Bradyrhizobium* (Ledermann et al. 2015). Transposon-mediated random transposition and site-specific homologous recombination are used to incorporate the target gene into the chromosome. However, such gene integration into the chromosome could cause undesirable effects on bacterial viability depending on the location of transposon insertion. Therefore, in this study, the fluorescent protein genes were inserted into the chromosome of the rhizobium by site-specific homologous recombination. As a target for the integration, we selected the downstream region of *nifX*, a gene conserved among rhizobium species and is least likely to be involved in the competitiveness of rhizobia. In fact, *nifX* is hardly expressed in the free-living state and is highly expressed only during the symbiotic nitrogen-fixing activity (Pessi et al. 2007). Our results revealed that the integration of *ZsGreen* and *DsRed* genes in this region did not affect bacterial growth (Fig. 1b) and symbiotic properties (Fig. 2). However, constitutive expression of tdTomato had slight negative effects on rhizobial performance. Since tdTomato itself is not cytotoxic to bacteria (Barbier and Damron 2016; Kong et al. 2016), a slight reduction in rhizobial performance may be an effect of overexpression of tdTomato, a tandem dimer of fluorescent protein of higher molecular weight than other fluorescent proteins (Shaner et al. 2005). Soybean nodules, on the other hand, tend to have a slight green autofluorescence. Taken together, based on our evaluation of fluorescent-labeled strains, we propose that the tdTomato-labeled strain is the most suitable for observation of infection dynamics in a single inoculum, as it shows the brightest fluorescence intensity in the red series. ZsGreen and DsRed-labeled strains is better to use for evaluation of their ability to compete for infection as they showed similar infectivity (Fig. S3).

In this study, we established the microscope observation system combining fluorescent rhizobia with RhizoFrame for nondestructive real-time imaging of root nodule symbiosis in the soil. The rhizotron is known as a representative device for nondestructive observation of root elongation dynamics at the macro level (Bengough et al 2016; Huck and Taylor 1982; Neufeld et al. 1989; Zhao et al. 2022). We have constructed Rhizoframe optimized for stereomicroscopic observation by adapting the rhizotron to the size of the observation stage of a stereomicroscope and reducing the thickness of the soil. These modifications allow the observation of soil-root-microbe interactions at the microscopic level. Although the reduction in rhizosphere volume due to thinner soil may have led to a reduction in the number of nodules in RhizoFrame (Fig. S5b), each nodule developed normally (Fig. S5a). These results suggest that RhizoFrame does not markedly suppress root nodule symbiosis, allowing visualization of rhizobial attachment onto soil and root hairs, infection thread formation, rhizobial colonization of host cells, and nodule development while retaining temporal-spatial information.

Soil comprises rocks, minerals, sediment, and organic matter (Carson et al. 2009; Uroz et al. 2015). This mosaic composition provides a variety of microhabitats for microorganisms (Hemkmeyer et al. 2018). In this study, we used vermiculite as the main soil carrier to be placed in RhizoFrame. Vermiculite is known to be an excellent carrier of rhizobia (Sparrow and Ham 1983), in fact, formation of biofilms of rhizobia on vermiculite surface has been reported (Fujishige et al. 2006). RhizoFrame system showed the localization of fluorescent-labeled rhizobia on vermiculite particles and root hairs in contact with them (Fig. 4b, c). Simultaneous visualization of soil, root, and fluorescent rhizobia is one of the advantages of our system, which can be used to understand how soil type affects rhizobial infection.

Prior to infection, rhizobia colonize the root surface. Some of these rhizobia form microcolonies, the starting points for forming infection threads (Fig. 5a indicated by arrows, 5b). In particular, large microcolonies (several µm in diameter) tend to form in the vicinity of epidermal cells containing infection threads (Figs. 5b, S7a, d). Following the colonization, many short infection threads within short root hair cells were formed in soybean roots (Figs. 5a, b, d, S7a–c). Their morphologies differ from those observed in *L. japonicus* (Fig. 6a) and those reported in other legumes such as *M. truncatula*, in which long infection threads grew through root hairs (Fournier et al. 2008; Liu et al. 2019; Rae et al. 2021). It has been reported that rhizobia infect soybean cells through infection threads from microcolonies trapped between short root hairs and epidermal cells (Rao and Keister 1978; Turgeon and Bauer 1985). The infection thread formation process we observed is likely the primary infection style in the soybean-*Bradyrhizobium* interaction. Although many infection threads were formed on the soybean roots in the early infection stage, most failed to guide rhizobia toward host cells, and no nodule primordia were induced under such infection threads. Only a few infection threads successfully penetrated nodule primordial cells, forming root nodules (Figs. 5d, S7d). It has been reported that the number of infection threads is much higher than the number of nodules formed on host plants (Gage 2004; Nishida et al. 2018), and our observations confirmed the same. In some cases, the formation of nodule primordia ceased after initial cell division (Fig. 4a; open arrowhead, Movie S1). In root nodule symbiosis, autoregulation of nodulation (AON) is one of the major negative regulatory systems controlling the number of nodules (Li et al. 2022; Oka-Kira and Kawaguchi 2006). Because photosynthetic carbon products are used as an energy source for symbiotic nitrogen fixation, AON maintains the balance between the benefits of gaining a nitrogen source and the cost of losing carbon (Nishida and Suzaki 2018). AON is driven by the rhizobial infection and suppresses further nodulation by blocking the infection thread penetration and cortical cell division. The phenomena observed in this study, such as the arrest of infection thread penetration and failure of nodule primordia formation, may be a part of AON that prevents excessive nodulation. Future studies of AON-related mutants and transgenic plants expressing AON-related reporter genes using RhizoFrame system would help understand whether these observations are related to AON.

In observing soybean roots, we found infection threads at a high frequency in slightly browning epidermal cells. The browning is visible in a bright field and can be a marker for finding cells containing infection threads under the fluorescent field. There were many cases in which the cell wall portion of epidermal cells could be clearly observed due to browning (Figs. 5a, b, S7). The browning of cells associated with rhizobial infection has been reported earlier (Vasse et al. 1993). In this case, the browning of cortical cells near epidermal cells containing infection threads is associated with the accumulation of defense response-related substances and suppresses the rhizobial infection (Vasse et al. 1993). In contrast, when we traced nodules from the infection thread formation stage, all nodules (*n* = 11) were originated from the infected threads in browning cells. This suggests that the browning of epidermal cells in soybean roots may result from a different mechanism than the defense response involved in suppressing rhizobial infection (Vasse et al. 1993). During the infection process of rhizobium-legume symbiosis, cell wall-degrading enzymes released by rhizobium promote modification of the host cell wall, leading to the formation of root nodules (Gage 2004; Mateos et al. 2001; Roy et al. 2020). Under the microcolonies we observed, such cell wall modification might have occurred locally at the site of contact with root epidermal cells. Browning of epidermal cells was also observed throughout the nodule primordium region containing infected threads that were successfully infected (Figs. 5d; 12 dai, 14 dai, S7d). Taken together, these observations suggest that browning in epidermal cells may be a response associated with cell wall remodeling during nodule development.

Mixed inoculation of soybeans with ZsGreen and tdTomato strains using RhizoFrame visualized the infection process of a single nodule with two strains (Fig. 5d). In this case, each rhizobium entered the host cells through distinct infection threads. In alfalfa and vetch, the formation of a single infection thread containing different bacterial strains has been reported (Gage 2002; Gage et al. 1996; Stuurman et al. 2000). In our study, almost all microcolonies and infection threads observed on the root epidermis were those formed by single-strain fluorescent rhizobia (Figs. 5a, b, d, S7). These results are consistent with previous observations in soybean (Ledermann et al. 2015) and may be a characteristic of rhizobium infection of soybean. Continuous imaging in RhizoFrame system revealed a difference in the colonization speed of two different strains in the nodule (Fig. 5d). In the natural soils, a variety of rhizobia are present and always compete for infection of host plants. Elucidating the mechanisms of such competitive infection will deepen our understanding of the evolution of rhizobial symbiotic ability and contribute to developing rhizobial materials that can overcome the infection competition from indigenous rhizobia and provide high inoculum efficacy. In addition to the conventional evaluation of symbiotic capacity by inoculation with a single rhizobium, recent studies have focused on the competitive analysis of rhizobial strains through root nodule symbiosis from synthetic communities of the rhizobia (Burghardt et al. 2018) and from mixed inoculation of gene-cassette, or fluorescent-labeled rhizobia (Bellabarba et al. 2021; Mendoza-Suarez et al. 2020). These methods help identify the rhizobia that infect and occupy individual nodule. Our RhizoFrame system, on the other hand, can capture the infection competition on the root surface in a nondestructive and continuous manner. Co-inoculation of several different fluorescent-labeled rhizobia in our system has the advantage of revealing at what point in the infection process the infection competition occurs.

The observation of transgenic *L. japonicus* expressing auxin-responsive reporter genes using RhizoFrame system showed root auxin dynamics synchronized with rhizobial infection in real time (Fig. 6d). Although some studies have reported the involvement of auxin in nodulation (Hirsch et al. 1989; Plet et al. 2011; Rightmyer and Long 2011; Wasson et al. 2006), the timing and site of auxin action during nodule development were poorly characterized. The creation of reporter plants has advanced our understanding of the relationship between auxin accumulation patterns and nodule development (Suzaki et al. 2012). Consistent with the previous report, strong auxin accumulation in a small number of cells in the vicinity of infection threads and actively dividing cortical cells was observed via RhizoFrame system (Fig. 6d, Movie S2). These results indicate that RhizoFrame system allows for a real-time and nondestructive reporter assay in the soil, a more natural condition. Previous studies have identified various genes that regulate root nodule symbiosis (Oldroyd 2013; Roy et al. 2020). Our system helps obtain a deeper understanding of how and when those genes act during nodulation.

Live imaging by fluorescent stereomicroscope using RhizoFrame system will allow us to understand soil–root–microbe interactions better, for example, tracking the infection process of parasitic microbes and the resulting changes in root morphology. However, because fluorescent labeling is required for visualization of microbes using RhizoFrame system, how to observe the behavior of microbes that are difficult to transform is an important task. We expect that further ideas to visualize the previously hidden interactions of soil, roots, and microorganisms will expand the further possibilities of the RhizoFrame system.

## Supplementary Information

Below is the link to the electronic supplementary material.

Supplementary file1 (PDF 5290 KB)

Supplementary file2 (MP4 2712 KB)

Supplementary file3 (MP4 8557 KB)

## Data Availability

The data underlying this article are available in the article and in its online supplementary material.
