# Supplementary material for: Rhizosphere frame system enables nondestructive live-imaging of legume-rhizobium interactions in the soil
Source: J Plant Res. 2023 Jul 4;136(5):769–80. doi: 10.1007/s10265-023-01476-2 (PMC10421814; doi:10.1007/s10265-023-01476-2)
Supplement: Supplementary file 1 — Supplementary file1 (PDF 5290 KB) [file 10265_2023_1476_MOESM1_ESM.pdf]

Supplementary Information

Title:
Rhizosphere Frame System Enables Nondestructive Live-Imaging of Legume-Rhizobium Interactions in the Soil

Journal:
Journal of Plant Research

Authors:
Hanna Nishida, Yoshikazu Shimoda, Khin Thuzar Win, and Haruko Imaizumi-Anraku

Corresponding author:
Haruko Imaizumi-Anraku
Institute of Agrobiological Sciences, National Agriculture and Food Research Organization
3-1-3 kannondai, Tsukuba, Ibaraki, 305-8604, Japan
Tel & Fax: +81-29-838-8377
Email: onko@affrc.go.jp

Table S1 Primers used in this work

Cloning of the *BjgroEL4* promoter in the EcoRI site of pK18mobsacB vector by In-Fusion reaction. (Underlines in Rv primer is the sequence that overlaps with fluorescent protein genes.)	
groEL4 promoter_Fw	5'–ACATGATTACGAATTCGAGCTCCACCGCGGTGGCGGCCGCAAACTGATAATCCATCT–3'
groEL4 promoter_Rv	5'–AGTTAATTTCTCCTCTTTAATGAATTCACGGGTGAGGCTGAAATAG–3'
Cloning of the fluorescent protein genes in the EcoRI site of pK18mobsacB vector by overlapping with the *BjgroEL4* promoter using In-Fusion reaction. (Underlines in Fw primers are the sequences that overlap with the *BjgroEL4* promtor. Underlines in Rv primers are the TrpA teminator sequences.)	
ZsGreen_Fw	5'–TTAAAGAGGAGAAATTAACTATGGCCCAAAGCAAACATGG–3'
ZsGreen_Rv	5'–TACCGAGCTCGAATTCTAAAAAAAAGCCCGCTCATTAGGCGGGCTAGGGCGAATTGGGTACCCGCTAGGGGAGAGCAGAGCCGG–3'
DsRed _Fw	5'–TTAAAGAGGAGAAATTAACTATGAGAGGATCTCACCATCA–3'
DsRed _Rv	5'–TACCGAGCTCGAATTCTAAAAAAAAGCCCGCTCATTAGGCGGGCTAGGGCGAATTGGGTACCCGCTACAGGAACAGGTGGTGGC–3'
tdTomato_Fw	5'–TTAAAGAGGAGAAATTAACTATGGTGAGCAAGGGCGAGGA–3'
tdTomato_Rv	5'–TACCGAGCTCGAATTCTAAAAAAAAGCCCGCTCATTAGGCGGGCTAGGGCGAATTGGGTACCCGCTACTTGTACAGCTCGTCCA–3'
Cloning of the downstream region of *nifX* in the XbaI site of pK18mobsacB vector by In-Fusion reaction.	
nifX_down-F	5'–CCGGGGATCCTCTAGGATGATATGACCGCAAAAATA–3'
nifX_down-R	5'–GCAGGTCGACTCTAGATCCCCTTCGCTCATGCA–3'
Verification of correct integration of ZsGreen into *nifX* downstream region.	
ZsGreen_check-F	5'–CGGATGCCAAGAACCAGAAG–3'
Verification of correct integration of DsRed into *nifX* downstream region.	
DsRed_check-F	5'–ACACCATCGTGGAGCAGTAC–3'
Verification of correct integration of tdTomato into *nifX* downstream region.	
tdTomato_check-F	5'–TGTTCCTGTACGGCATGGAC–3'
Verification of correct integration of plasmids into *nifX* downstream region, binds to chromosomal region	
nifX_down_check-R	5'–ATTGACCTGATCCAGCGGG–3'
Primer set 1 for identification of rhizobia in nodules. The fagment (1.1 kbp) is detected in USDA110 WT.	
Identification 1_F	5'–GCGATCGTTTGAGTTCGACG–3'
Identification 1_R	5'–ATTGACCTGATCCAGCGGG–3'
Primer set 2 for identification of rhizobia in nodules. The fagment (0.6 kbp) is detected in fluorescent strains.	
Identification 2_F	5'–AGAAAGGCGGACAGGTATCC–3'
Identification 2_R	5'–GGTGGAGCTCGAATTCGTAATC–3'

> ZsGreen (693bp)

ATGGCCCAAAGCAAACATGGCCTCACCAAGGAGATGACGATGAAGTATCGGATGGAAGGCTGTGTAGATGGGC
ACAAGTTCGTCATCACAGGTGAGGGCATTGGCTATCCGTTTAAGGGCAAACAGGCGATCAACCTTTGCGTCGT
GGAAGGAGGTCCGTTGCCGTTTGCGGAAGACATCCTGTCAGCGGCCTTCAACTATGGCAATCGGGTCTTCACC
GAGTATCCCCAAGACATCGTCGACTACTTCAAGAACTCGTGTCCAGCAGGGTATACGTGGGATCGCAGCTTCC
TGTTCGAAGACGGCGCTGTCTGCATCTGCAATGCCGACATCACGGTGAGTGTCGAGGAGAATTGCATGTACCA
CGAGTCGAAGTTCTACGGGGTGAACTTTCCGGCAGATGGACCGGTGATGAAGAAGATGACCGATAACTGGGAG
CCTTCGTGCGAGAAGATCATTCCCGTTCCGAAGCAGGGCATCCTCAAAGGCGACGTGAGCATGTACCTACTGC
TGAAAGACGGCGGTCGTTTGCGCTGCCAGTTCGACACGGTCTACAAGGCGAAGTCCGTTCCACGCAAGATGCC
GGATTGGCACTTCATCCAGCACAAACTGACTCGAGAAGACCGCTCGGATGCCAAGAACCAGAAGTGGCATCTC
ACCGAGCATGCCATAGCGTCCGGCTCTGCTCTCCCCTAG

Fig. S1 Nucleotide sequence of the codon-optimized *ZsGreen* gene

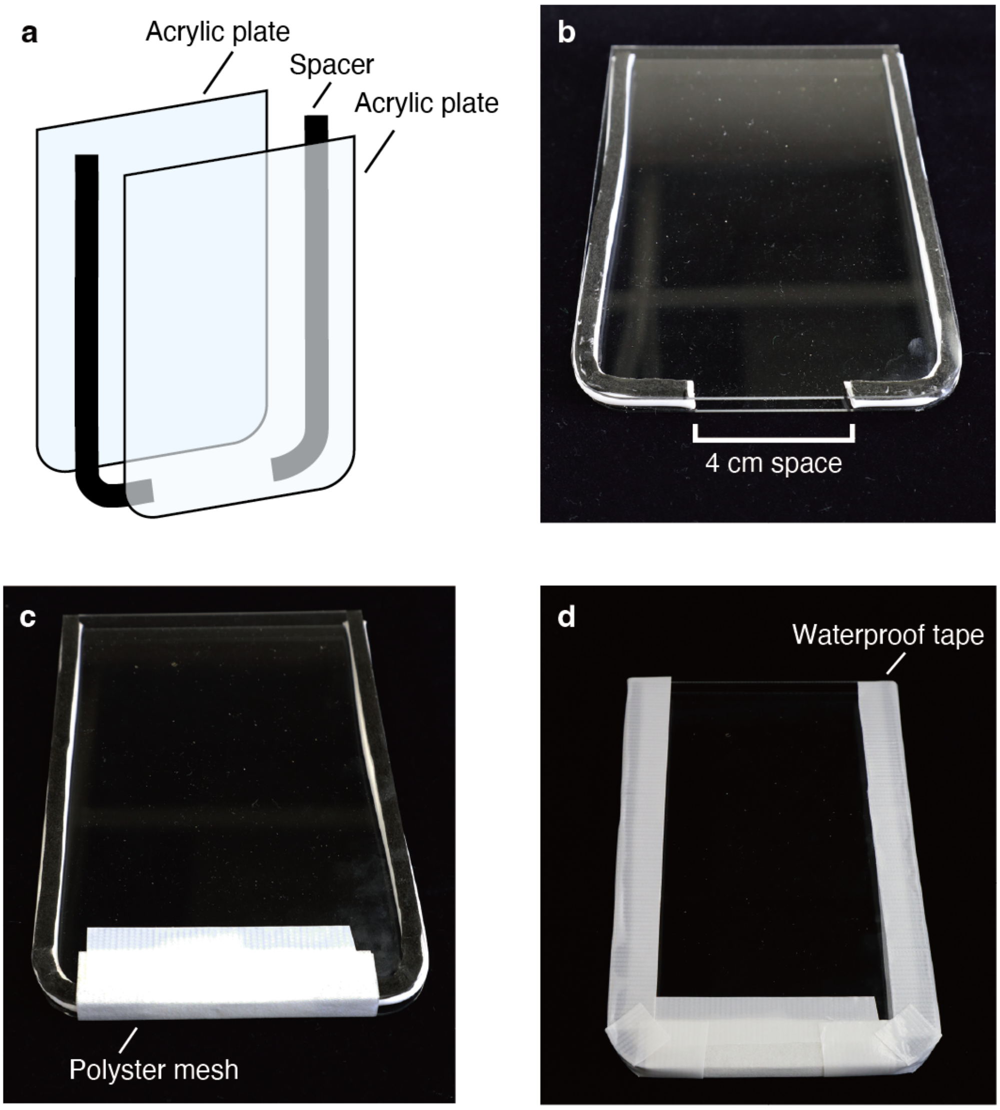

Fig. S2 How to make RhizoFrame with commercially available materials

Materials

Transparent acrylic plates: 100 mm wide, 1 mm thick, and 150 mm high, custom-made. The bottom corners are rounded off (R = 15.0 mm) as necessary.

Spacer: 6 mm wide and 3 mm thick, NICHIAS SOFT SEAL (NICHIAS Co.)

Water-resistant double-sided tape: 5 mm wide and 0.8 mm thick, High Tack Double-Sided Adhesive Tape (3M Japan Limited)

Polyester mesh: 38 µm aperture, cut into 5 × 4 cm

Waterproof tape: Ace Cloth 011 Single-Sided Airtight Waterproof Tape (Koyo Chemical)

Assembly of RhizoFrame with commercially available material

(a and b) Two spacers are attached with double-sided tape, as shown in the figure to create about 4 cm space at the bottom. Then, another acrylic plate is stacked on top to create about 5 mm space. (c) A polyester mesh is attached to the bottom of the acrylic plate with a 5 cm-wide waterproof tape to cover the 4 cm space. (d) The sides of the assembled RhizoFrame are covered with waterproof tape.

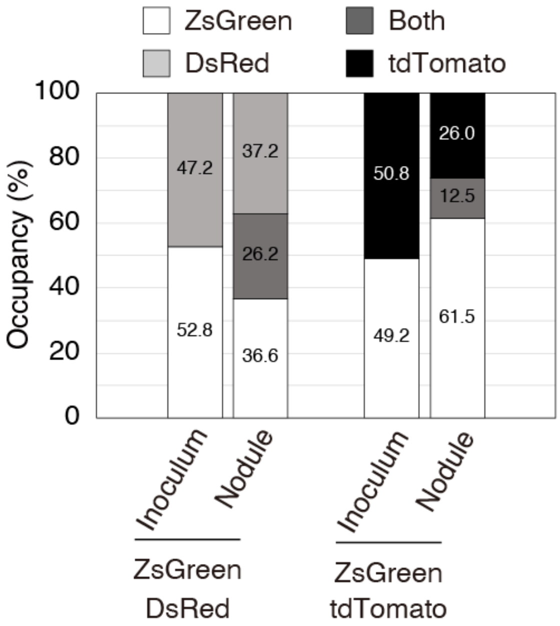

Fig. S3 Nodule occupancy during co-inoculation of ZsGreen with DsRed (n = 5 plants, 191 nodules) or tdTomato (n = 4 plants, 96 nodules). Enrei was inoculated with approximately 1:1 mixture of ZsGreen and DsRed or tdTomato, and after 21 days, the number of nodules colonized by each strain was measured. Each strain in nodules was identified by PCR. “Inoculum” indicates the ratio of colonies formed by rhizobia on agar after mixture was plated (n = 667–756 colonies).

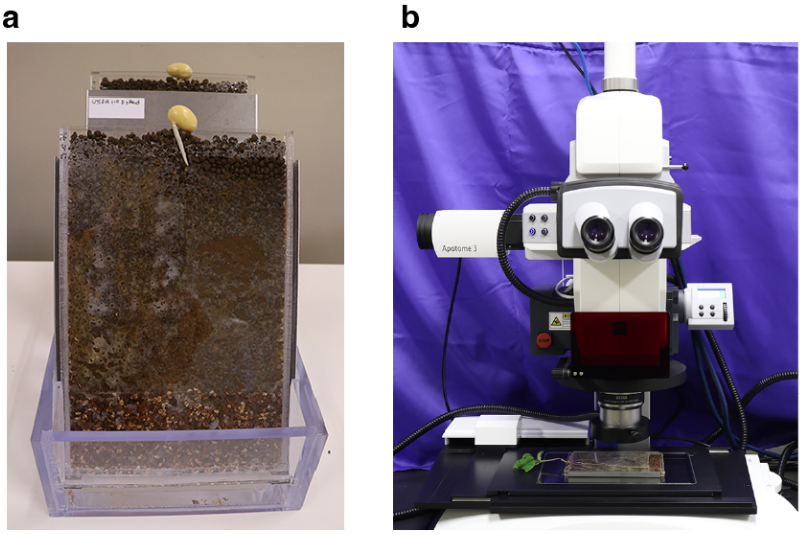

Fig. S4 Cultivation of plants using RhizoFrame and observation with a microscope. (a) Two-day-old soybean seedlings were transplanted into RhizoFrame. (b) Observation of RhizoFrame using a fluorescent microscope.

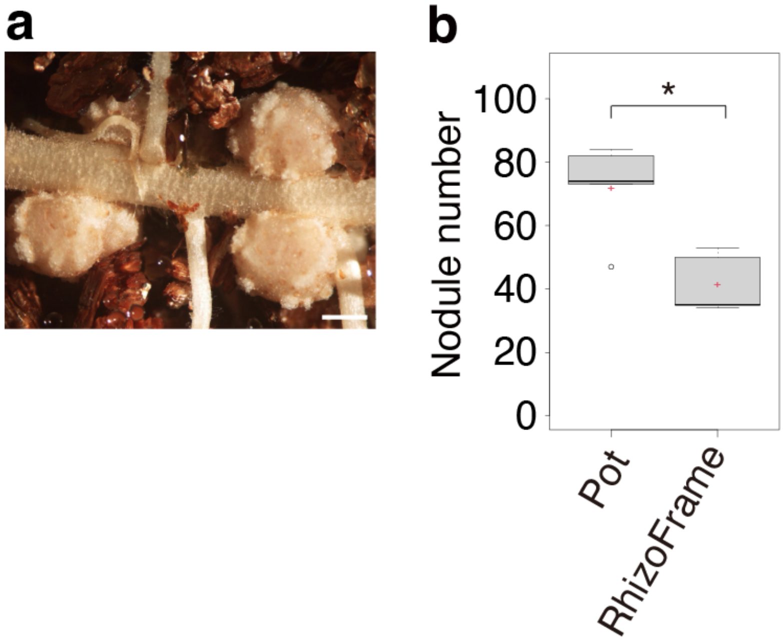

Fig. S5 (a) Nodule formation and (b) nodule number (n = 5 plants) at 21 days after inoculation in RhizoFrame. Soybeans cv. Enrei were inoculated with *B. diazoefficiens* USDA 110 WT. Scale bars = 1 mm (a). Centerlines in the boxplots show the medians, and upper and lower quartile limits are shown as horizontal bars. Points represent outliers, and red crosses indicate the sample means. *P < 0.05 by a Wilcoxon rank sum test. Nodule number in pots is the same data as in Fig. 2b.

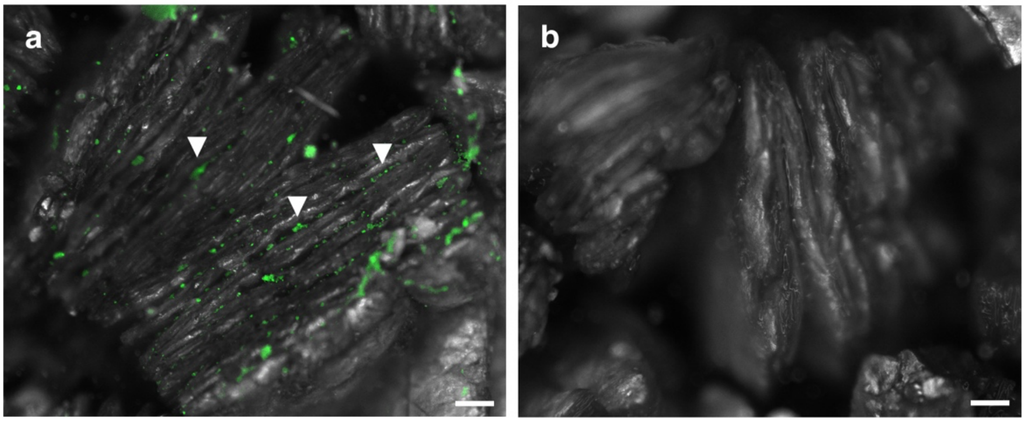

Fig. S6 Visualization of rhizobia on soil particles with RhizoFrame system. (a) Localization of rhizobia on the vermiculite (7 days after inoculation). Green fluorescence indicates the presence of rhizobia. (b) Fluorescence observation of non-inoculated vermiculite. We poured the B&D solution with (a) or without (b) ZsGreen strain over the vermiculite in RhizoFrame and observed it under the same conditions with a fluorescence microscope. Scale bars = 200 μm.

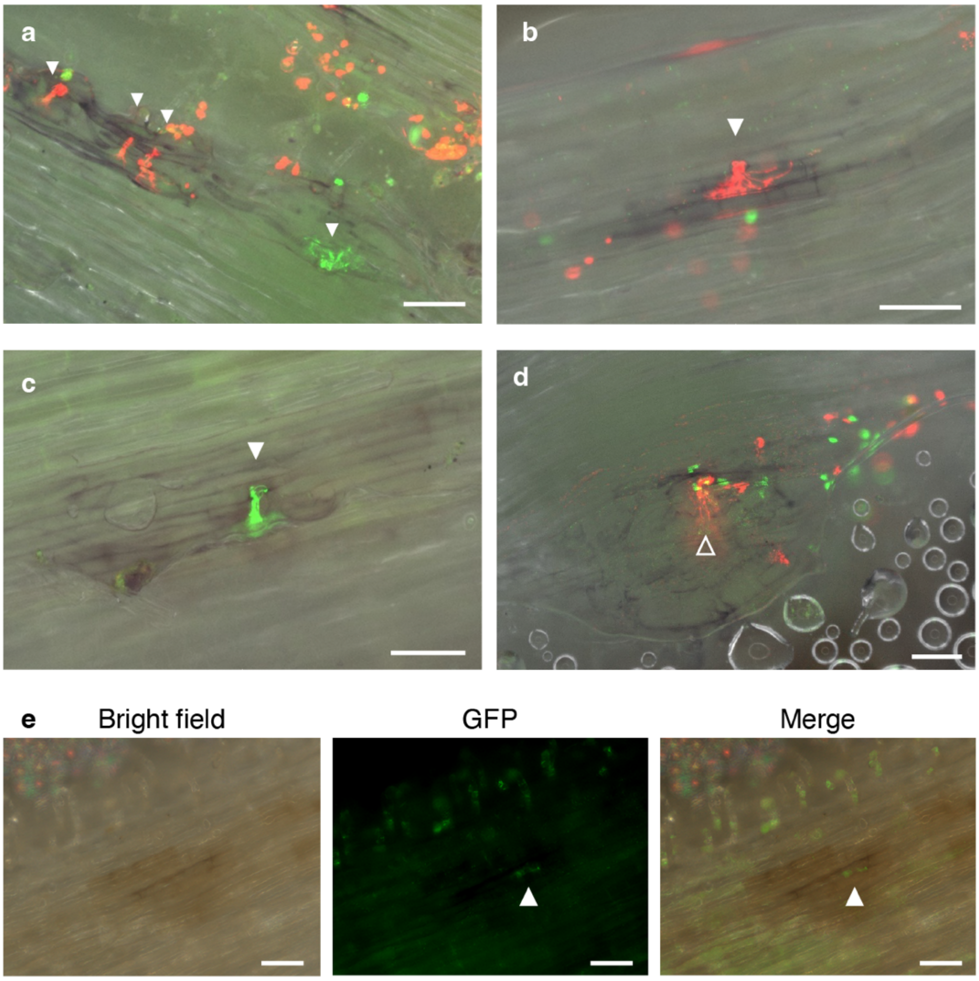

Fig. S7 Infection threads formed on soybean roots. Infection threads at 7 days after inoculation (dai; a, b, e) and 9 dai (c). Epidermal cells containing infection threads were pigmentated (arrowheads). (d) Infection threads (open arrowhead) that have successfully penetrated the dividing cortical cells at 10 dai. Pigmentation is seen in the epidermis. Soybean cv. Enrei was co-inoculation of strains ZsGreen and tdTomato (about 1:1 ratio; a–d) or inoculated with ZsGreen strain (e) and were observed under a fluorescence microscope. Green and red fluorescence indicate the presence of strains ZsGreen and tdTomato, respectively. Scale bars = 100 µm.

Movie S1 Time-lapse image of nodulation in soybean from 5 to 10 days after inoculation. Soybean cv. Fukuyutaka was inoculated with USDA 110 strains DsRed and was observed by a fluorescence microscope. Images were acquired 31 times every 4 hours. Scale bars = 1 mm.

Movie S2 Time-lapse image of auxin response patterns during nodulation from 6 to 8 days after inoculation. Auxin accumulation patterns were indirectly shown by GFP expression (green) in *DR5::GFP-NLS* MG20 plants. Red signals indicate the presence of *M. loti* MAFF303099 that constitutively expresses DsRed. Images were acquired 26 times every 4 hours. Scale bars = 500 μm.
